# Influence of Surgical Timing on the Risk of Permanent Pacemaker Implantation in Acute Aortic Valve Endocarditis †

**DOI:** 10.3390/medicina62081451

**Published:** 2026-07-27

**Authors:** Michele D’Alonzo, Lorenzo Di Bacco, Antonio Fiore, Massimo Baudo, Emmanuel Villa, Giovanni Troise, Thierry Folliguet, Claudio Muneretto

**Affiliations:** 1 Cardiac Surgery Unit, “Poliambulanza Foundation” Hospital, Via Bissolati 57, 25124 Brescia, Italy; emmanuel.villa@poliambulanza.it (E.V.); giovanni.troise@poliambulanza.it (G.T.); 2 Cardiac Surgery Unit, “Spedali Civili”, University of Brescia, 25123 Brescia, Italy; lorenzo.dibacco@hotmail.it (L.D.B.); claudio.muneretto@unibs.it (C.M.); 3 Cardiac Surgery Unit, Hôpital “Henri Mondor—Assistance Publique Hôpîtaux de Paris”, 94100 Créteil, France; antonio.fiore@aphp.fr (A.F.); thierry.folliguet@aphp.fr (T.F.); 4 Department of Cardiac Surgery Research, Lankenau Institute for Medical Research, Main Line Health, Wynnewood, PA 19096, USA; massimo.baudo@icloud.com

**Keywords:** aortic endocarditis, surgical timing, guidelines, permanent pacemaker, reinfection

## Abstract

*Background and Objectives*: The optimal timing for surgical intervention in acute aortic valve infective endocarditis (IE) remains debated. Although European guidelines distinguish emergency, urgent, and delayed indications, some centres postpone surgery, concerned that operating before sufficient antibiotic sterilization could increase IE relapse risk. *Materials and Methods*: This retrospective, observational, multicenter study included patients with acute aortic valve IE. Patients who were not operated, had non-aortic valve IE, or underwent emergency surgery were excluded. Surgery within 7 days of starting targeted antibiotics was classified as “early”; surgery between 7 and 30 days as “late.” Primary outcomes were in-hospital mortality and 30-day permanent pacemaker implantation. Secondary endpoints included reinfection, reoperation for IE relapse, and mid-term survival. *Results*: A total of 203 patients included: 104 early and 99 late. In-hospital mortality was comparable (early: 16 patients, 15.4%; late: 16 patients, 16.2%; *p* = 0.90). Permanent pacemaker implantation was higher in the late group (early: 9 patients, 8.7%; late: 18 patients, 18.2%; *p* = 0.046). Mid-term survival at four years was similar (early: 67.1 ± 5.8%; late: 59.7 ± 8.1%; *p* = 0.71). Recurrence of IE (early: 5.7%; late: 4.8%; *p* > 0.9) and reoperation for recurrent IE (early: 5.7%; late: 4.8%; *p* > 0.9) did not differ. *Conclusions*: Delaying surgery for acute aortic valve IE does not improve procedural safety, as in-hospital mortality is similar. Early intervention does not increase reinfection or reoperation risk, and may reduce permanent pacemaker implantation, likely by preventing progressive fibrosis of the cardiac conduction system caused by infection and prolonged antibiotic exposure.

## 1. Introduction

Infective endocarditis (IE) remains an uncommon yet clinically significant disease, with an estimated annual incidence of 3–10 cases per 100,000 individuals [1]. Although relatively rare, epidemiological evidence suggests a progressive increase in its occurrence and a persistent underreporting of cases [2]. Despite improvements in diagnostic modalities and antimicrobial therapy, IE continues to carry a substantial risk of morbidity and mortality. In this context, surgery plays a central role, particularly in the treatment of aortic valve endocarditis, where operative management can be decisive for infection eradication [3].

The indication and timing of surgery in IE represent one of the most challenging aspects of management. Surgical intervention is often required when infection extends to peri-annular tissues or when hemodynamic instability ensues. Yet, these procedures are technically demanding, and patients are frequently critically ill. Although specific prognostic models have been developed to estimate postoperative mortality in IE [4], their use in routine clinical practice remains limited, with decision-making often guided by broader cardiac surgery risk scores and expert judgment.

Nevertheless, the optimal timing of surgery in IE remains one of the most debated issues in contemporary practice. The 2023 European endocarditis guidelines [5] recommend a stratified approach, distinguishing between emergency surgery (within 24 h), urgent surgery (within 3–5 days), and delayed surgery (following a short antibiotic course with close monitoring). However, due to the heterogeneity of clinical presentations and the ethical and logistical challenges of randomized trials, most evidence derives from observational retrospective studies.

Consequently, decisions regarding surgical timing are often guided by institutional protocols, the individual experience of the surgical team, and patient-specific factors such as age, comorbidities, and the extent of the infectious process. This approach, however, may compromise the consistency and objectivity of clinical decision-making. Interestingly, data from pediatric cohorts suggest that early surgery may be both safe and advantageous, challenging the traditional notion of delaying surgical intervention [6]; nevertheless, similar benefits have not been consistently demonstrated in the adult population.

An area that remains insufficiently explored concerns the need for permanent pacemaker implantation following valve surgery for IE. While early pacemaker implantation after surgical aortic valve replacement has been associated with increased long-term mortality [7], more recent evidence by Bearpark et al. reported no association between pacemaker implantation and adverse outcomes, such as mortality, heart failure, or reinfection, in patients with infective endocarditis [8]. However, the potential influence of surgical timing on this postoperative complication has not been systematically investigated.

Considering these gaps, the present study aims to test the hypothesis that performing surgical intervention within seven days of targeted antibiotic therapy may influence the incidence of permanent pacemaker implantation among patients with acute aortic valve endocarditis.

## 2. Materials and Methods

### 2.1. Study Design and Ethical Considerations

This was a retrospective, observational, multicenter analysis conducted to assess the outcomes of patients undergoing cardiac surgery for acute infective endocarditis (IE), with a particular focus on cases involving the aortic valve. The study complied with the principles of the Declaration of Helsinki. Written informed consent had been obtained from all patients at the time of their inclusion in institutional databases for scientific purposes. Formal ethical approval was not required due to the retrospective, observational and non-interventional nature of the study, according to applicable regulations. All data were anonymized prior to analysis to ensure patient confidentiality.

### 2.2. Patient Population and Data Collection

The study included patients diagnosed with acute aortic valve infective endocarditis between January 2016 and March 2024. Data were collected from institutional databases of three participating centres: Spedali Civili (Brescia, Italy), Poliambulanza Foundation (Brescia, Italy), and Henri Mondor (Créteil, France).

Preoperative demographic and clinical data were recorded, including age, gender, major comorbidities, and presenting symptoms at admission. The diagnosis of IE was established on the basis of clinical criteria, laboratory evidence, and imaging studies—transthoracic and transesophageal echocardiography, and, when indicated, additional modalities such as computed tomography (CT), positron emission tomography (PET), or magnetic resonance imaging (MRI). Intraoperative findings further supported the diagnosis. Targeted antibiotic therapy was defined as an antimicrobial regimen selected according to blood culture results and antimicrobial susceptibility testing.

Exclusion criteria included emergency surgery (defined as surgery performed within 24 h of antibiotic therapy), age under 18 years, absence of infection involving the aortic valve, and medical management without surgery.

### 2.3. Definitions, Study Endpoints, and Follow-Up

After application of inclusion and exclusion criteria, a total of 203 patients were included in the final analysis. The study cohort was divided into two subgroups: EARLY group: patients who underwent surgical intervention within 7 days from the start of targeted antibiotic therapy; LATE group: patients who underwent surgery after at least 7 days of antibiotic therapy but within 30 days, thereby excluding chronic or late-stage IE cases.

The primary endpoints were in-hospital mortality and the 30-day incidence of permanent pacemaker implantation. Secondary outcomes included reinfection, reoperation due to infection, and mid-term survival.

Patient follow-up was completed in October 2024, with a mean duration of 657 days. Follow-up information was obtained through outpatient evaluations or structured telephone interviews when in-person visits were not feasible.

### 2.4. Statistical Analysis

The normality of continuous variables was assessed using the Kolmogorov–Smirnov test. Continuous variables were compared using a two-tailed independent Student’s *t*-test when normally distributed, and by the Mann–Whitney U-test when not normally distributed. Categorical variables were analyzed with the chi-square (χ^2^) or Fisher’s exact test, as appropriate. Differences in survival between groups were analyzed using the Kaplan–Meier method, with comparisons made via log-rank testing. A *p*-value ≤ 0.05 was considered statistically significant.

Firth bias-reduced logistic regression was applied to evaluate predictors of pacemaker rates. The variance inflation factor (VIF) was measured for every variable in the regression model to test multicollinearity. A value greater than 5 indicates potentially severe correlation between a given predictor variable and other predictor variables in the model.

Data extraction was performed using Microsoft Excel (Microsoft, Redmond, WA, USA), and statistical analyses were carried out using R software (version 4.3.1; R Foundation for Statistical Computing, Vienna, Austria) within RStudio (version 2024.09.1).

## 3. Results

Of the 203 patients included in the study, 104 were classified into the EARLY group, while 99 were assigned to the LATE group. Baseline demographic and clinical characteristics were comparable between the EARLY and LATE groups (Table 1). No significant differences were observed regarding age, gender, comorbidities, or preoperative clinical status. However, patients in the EARLY group were more frequently affected by native aortic valve endocarditis (72% vs. 59%, *p* = 0.04) and exhibited slightly higher preoperative eGFR values (77.5 ± 33.8 vs. 69.2 ± 31.1 mL/min/1.73 m^2^, *p* = 0.04). As expected, the delay between antibiotic initiation and surgery was significantly shorter in the EARLY group (median 3 vs. 14 days, *p* ≤ 0.001).

**Figure 1:**
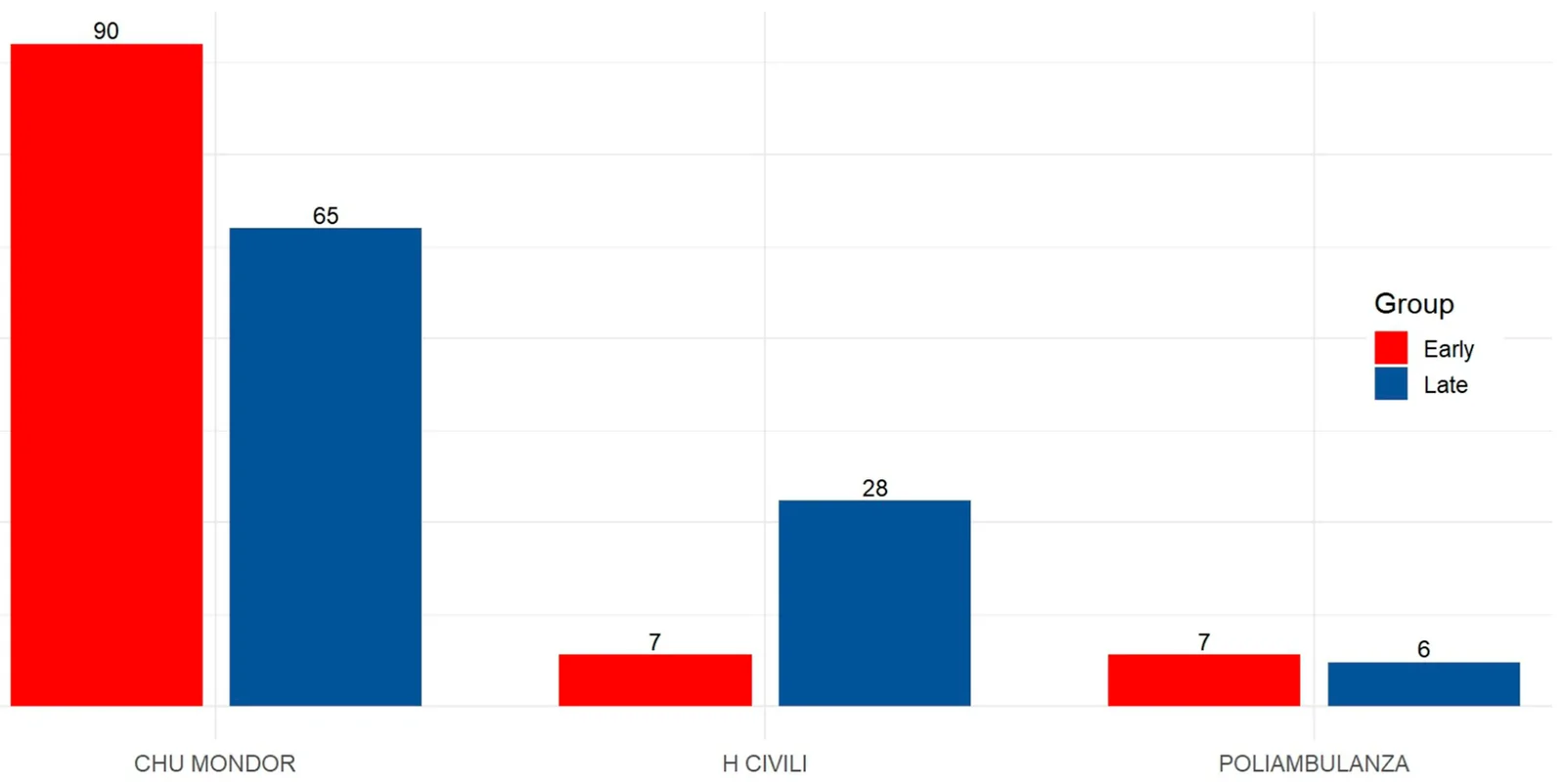
Distribution of patients by center and surgical timing group.

Figure 1 shows the distribution of patients across centres: a clear predominance of cases comes from CHU Mondor, which enrolled the majority in both groups. The figure also reflects distinct institutional approaches to surgical timing: CHU Henri Mondor shows a clear tendency toward early surgical intervention, with most of its patients belonging to the EARLY group (90 vs. 65 patients). In contrast, at Spedali Civili, the predominant approach is delayed surgery, as shown by the higher number of LATE cases (28 vs. 7 patients). Fondazione Poliambulanza demonstrates a more balanced or intermediate strategy, with a comparable number of patients treated early and late.

Operative data were similar between the two groups (Table 2). The majority of patients underwent isolated aortic valve replacement (84%), with no significant difference between EARLY and LATE groups (88% vs. 80%, *p* = 0.14). Complex procedures such as Bentall operations, concomitant mitral valve surgery or coronary surgery were evenly distributed. Cardiopulmonary bypass and aortic cross-clamp times were also comparable between groups, suggesting similar surgical complexity. A trend towards a higher use of Homografts was observed in the LATE group (9.1% vs. 2.9%, *p* = 0.061).

Early postoperative outcomes are summarized in Table 3. Overall, in-hospital mortality was comparable between groups (EARLY 15.4% vs. LATE 16.2%, *p* = 0.9). Similarly, the need for mechanical circulatory support, either with IABP or ECMO, was low and comparable between cohorts. The incidence of major postoperative complications such as prolonged mechanical ventilation, stroke, and reoperation for bleeding did not differ significantly. A trend toward a higher rate of postoperative atrial fibrillation was observed in the LATE group (34% vs. 23%, *p* = 0.076). Renal outcomes according to the KDIGO classification were evenly distributed, with approximately two-thirds of patients showing no postoperative renal impairment. Patients in the LATE group required blood transfusions more frequently (72% vs. 57%, *p* = 0.026) and had a significantly longer ICU stay (mean: 4.3 vs. 2.7 days, *p* = 0.003). At 30 days, the need for permanent pacemaker implantation was significantly higher in the LATE group (18.2% vs. 8.7%, *p* = 0.046), while rates of re-hospitalization and sternal wound infection were low and similar between groups.

Because the limited number of pacemaker events precluded a conventional multivariable model, surgical timing was further evaluated with a Firth bias-reduced logistic regression (Table 4). In this adjusted model, the LATE group remained independently associated with permanent pacemaker implantation (OR 2.51, 95% CI 1.07–6.21; *p* = 0.034), whereas none of the remaining covariates, including treatment center and native vs. prosthesis aortic valve, reached statistical significance. Multicollinearity was assessed through the variance inflation factor for each predictor, and no collinearity issues were identified (all VIF < 1.3).

**Figure 2:**
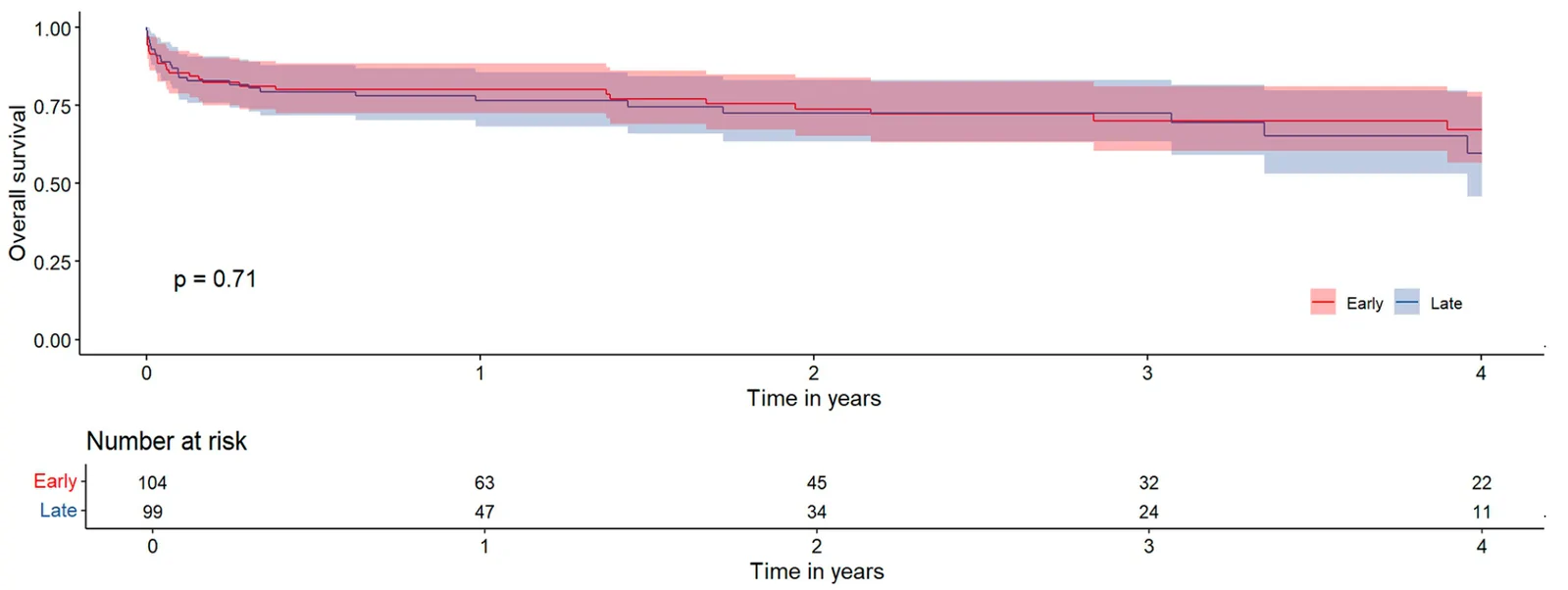
Kaplan–Meier survival curves comparing overall survival between EARLY and LATE groups. Shaded areas represent 95% confidence intervals.

Regarding late outcomes, overall mortality was comparable between groups. One-year survival was 80.0 ± 4.0% in the EARLY group and 76.5 ± 4.5% in the LATE group (*p* = 0.79), while four-year survival was 67.1 ± 5.8% and 59.7 ± 8.1%, respectively (*p* = 0.71) (Figure 2). Among the 171 patients alive at last follow-up (Table 5), redo operations specifically related to IE occurred in 9 patients (5.3%), without differences between groups (EARLY 5.7%, LATE 4.8%; *p* > 0.9). Recurrence of IE not requiring surgery was observed in 12 patients (7.0%), comparable between the two groups, while the overall rate of IE relapse was 12% (21 patients). Late strokes were infrequent, occurring in 5 patients (2.9%).

## 4. Discussion

The present study aimed to explore the impact of surgical timing on outcomes among patients undergoing surgery for acute aortic valve IE, focusing not only on in-hospital and mid-term mortality but also on procedure-related complications such as PPM implantation. Despite substantial advances in the medical and surgical management of IE, the optimal timing of surgery remains controversial. Historically, surgeons have often preferred to delay intervention in order to prolong preoperative antibiotic therapy, aiming to operate in a “cleaner” and more stable field, thereby minimizing operative risk and recurrence rates [9]. Indeed, one of the major concerns in IE management remains the risk of recurrence, which may necessitate high-risk reoperation.

In our cohort, after excluding emergency cases, where surgical delay is not an option, in-hospital mortality rates were comparable between the two groups, suggesting that an early surgical approach does not compromise short-term survival. Moreover, our analysis also demonstrated that surgical timing did not significantly influence other postoperative outcomes, such as the need for ECMO, renal failure, or prolonged mechanical ventilation. This finding challenges the long-held belief that delaying surgery improves procedural safety by allowing infection control through extended antibiotic treatment. It should be stressed, however, that the study was not designed or powered as a non-inferiority/equivalence study; the comparable rates therefore represent absence of evidence of a difference rather than evidence of equivalence, and a type II error cannot be excluded.

A particularly intriguing finding of our analysis concerns the incidence of PPM implantation. The atrioventricular node and the proximal His bundle lie in close anatomical relationship with the aortic annulus, particularly at the level of the membranous septum and the commissure between the right and non-coronary cusps, which makes the conduction system especially vulnerable in aortic valve endocarditis. Several, partly additive, mechanisms may lead to permanent pacemaker dependency in this setting: direct infectious extension with peri-annular abscess and tissue destruction; oedema and acute inflammation of the peri-nodal tissue; intraoperative mechanical or thermal injury during debridement and prosthesis implantation, the risk of which increases with the extent of annular involvement; and progressive fibrotic remodeling of the conduction tissue.

The rate of PPM implantation was nearly twice as high in the LATE group compared with the EARLY group. This observation invites speculation regarding the underlying pathophysiological mechanisms. Our hypothesis is that conduction system injury in aortic valve IE results not only from direct infectious destruction of the conduction pathways but also from inflammatory and fibrotic processes induced or perpetuated by prolonged antibiotic therapy. Thus, by performing surgery earlier, the propagation of damage may be interrupted, potentially preserving conduction function. Nevertheless, even within the EARLY group, the incidence of PPM implantation remained markedly higher than that typically reported after standard aortic valve replacement for non-infective indications, a difference likely attributable to the prolonged postoperative antibiotic therapy (usually extending for 4–6 weeks), which may contribute to inflammatory and fibrotic alterations affecting the cardiac conduction system. We hypothesize that a longer interval of active peri-annular infection and sustained systemic inflammation before surgery, as occurs in a deliberate antibiotic-first delay, may serve to facilitate the transition from potentially reversible oedema/inflammation to irreversible fibrosis, thereby increasing the likelihood of permanent block. Conversely, earlier source control might interrupt this process. We emphasize that this mechanistic interpretation is speculative and hypothesis-generating only, as no histopathological, electrophysiological, or imaging data are available to support it.

This comparison is particularly relevant in light of data from Glaser [10], who analyzed 24,938 patients undergoing primary surgical aortic valve replacement for non-IE indications between 1997 and 2018. In their cohort, postoperative PPM implantation occurred in only 3.4% of patients and was associated with increased all-cause mortality and heart failure risk. Similar findings were reported by Gerson et al., supporting the rationale for developing strategies aimed at minimizing the need for PPM implantation, especially in the IE population [7]. Previous literature suggests that the need for PPM implantation after aortic valve surgery is more strongly related to advanced preoperative valve disease than to pre-existing conduction abnormalities [11]. Indeed, risk factors for postoperative PPM include annular calcification, bicuspid aortic valve, female sex, preoperative right or left bundle branch block, prolonged cardiopulmonary bypass time, and hypertension [12]. It is also important to consider that patients with acute IE often require surgical intervention precisely because of conduction disturbances, and irreversible atrioventricular block demanding a permanent pacemaker is a frequent postoperative scenario in this setting.

In this context, the findings by Bearpark [8] are particularly noteworthy: among 2175 patients who underwent surgery for aortic valve endocarditis, 168 (8%) required permanent pacemaker implantation, a rate consistent with our EARLY group but lower than that observed in our LATE cohort. Interestingly, their study reported that PPM implantation after surgery for aortic valve IE was not associated with increased mortality, heart failure, or reinfection. While this is a valuable contribution, several limitations must be acknowledged, including the absence of microbiological data, information on pre-existing conduction abnormalities, and moreover, the long enrolment period (1997–2022) that spans significant changes in diagnostic protocols, antimicrobial therapy, and surgical practice, which could confound the interpretation of results.

Regarding mid-term mortality, our follow-up extending up to 4 years showed no significant difference in overall survival between the EARLY and LATE groups. Similarly, the rates of reinfection and redo surgery for recurrent endocarditis were comparable, suggesting that a prolonged course of preoperative antibiotic therapy does not confer additional benefit in preventing recurrence.

A recurrent limitation in existing literature is the tendency to generalize infective endocarditis as a single entity, without distinguishing between aortic and mitral valve involvement. The mitral valve, unlike the aortic valve, is often amenable to repair, and delaying surgery in stable patients may increase reparability rates without negatively affecting outcomes, as demonstrated by Dreyfus [13] in 1990 and later confirmed by Di Bacco [14]. In contrast, aortic valve repair remains uncommon, even in elective settings, and in the context of IE, valve replacement is almost invariably required. For this reason, we specifically focused on patients with acute aortic valve endocarditis while excluding cases where the primary indication for surgery involved other valves.

Another point of consideration is the assumption that prosthetic valve endocarditis carries a higher operative risk than native valve infection, leading to a more conservative, delayed surgical approach. However, several studies have demonstrated that perioperative outcomes are comparable between native and prosthetic aortic valve endocarditis [15,16]. Therefore, both patient groups were included in our analysis, as this variable was not expected to introduce significant confounding effects, unlike the inclusion of isolated mitral endocarditis cases, which represent a different surgical and prognostic entity.

Limitations. This study has several limitations. First, it is a retrospective, non-randomized analysis and, despite multivariable adjustment, residual and unmeasured confounding cannot be excluded. Second, there was substantial heterogeneity in surgical-timing strategy across the three participating centres (Figure 1): because timing and center are strongly associated, the two effects are difficult to fully disentangle, and the center-adjusted estimates should be interpreted with this near-collinearity in mind. Third, the study was not designed or powered as an equivalence/non-inferiority study and no a priori sample-size calculation was performed; non-significant comparisons (mortality, reinfection, reoperation) should therefore be read as absence of evidence rather than evidence of equivalence, and a type II error is possible, especially given the limited number of pacemaker events, which also constrained the number of covariates in the multivariable model. Fourth, by design, only operated patients were included; patients managed conservatively or who died while awaiting a planned delayed operation were not captured, which may introduce a selection/immortal-time bias affecting the LATE group. Fifth, the 7-day threshold, although clinically motivated, is to some extent arbitrary; although widely accepted in the current literature, it represents only a conventional cutoff that may not fully reflect individual clinical prioritization or the continuous spectrum of disease progression. Finally, granular data on pre-operative conduction status, microbiology, and pre-operative inflammatory markers were collected retrospectively and were incomplete for some patients (procalcitonin, in particular, was not systematically available), limiting the depth of the mechanistic analysis.

## 5. Conclusions

Delaying cardiac surgery for acute aortic endocarditis did not appear to improve procedural safety, as in-hospital mortality remains comparable. Early surgical intervention neither increases the risk of reinfection nor the need for late reoperation, while offering potential benefits such as a lower incidence of permanent pacemaker implantation. The reasons underlying this finding remain uncertain: this reduction may be attributed to the interruption of progressive fibrosis affecting the cardiac conduction system, which may result from both infection and prolonged antibiotic exposure. Further studies are warranted to clarify the mechanisms involved and to better define the optimal timing of surgery in this setting.

## Figures and Tables

**Table 1:** Preoperative characteristics.

Characteristic	Overall, N = 203	Early, N = 104	Late, N = 99	*p*-Value
Delay * (days)	7 [3, 14]	3 [2, 4]	14 [10, 24]	<0.001
Age	66.4 (12.4)	65.3 (13.0)	67.5 (11.6)	0.3
Gender (male)	166/203 (82%)	86/104 (83%)	80/99 (81%)	0.7
BSA	1.9 (0.2)	1.9 (0.2)	1.9 (0.2)	0.4
BMI	26.5 (5.6)	26.6 (5.5)	26.4 (5.7)	0.9
Drug abuser	3/203 (1.5%)	1/104 (1.0%)	2/99 (2.0%)	0.6
Hypertension	123/203 (61%)	58/104 (56%)	65/99 (66%)	0.15
COPD	17/203 (8.4%)	8/104 (7.7%)	9/99 (9.1%)	0.7
Dialysis	5/203 (2.5%)	3/104 (2.9%)	2/99 (2.0%)	>0.9
eGFR	73.5 (32.7)	77.5 (33.8)	69.2 (31.1)	0.044
Aortic Valve (native)	133/203 (66%)	75/104 (72%)	58/99 (59%)	0.043
NYHA advance (III-IV class)	68/203 (33%)	40/104 (38%)	28/99 (28%)	0.12
Nosocomial Infection	5/203 (2.5%)	2/104 (1.9%)	3/99 (3.0%)	0.7
Fever	157/203 (77%)	79/104 (76%)	78/99 (79%)	0.6
Large Vegetation (>10 mm)	167/203 (82%)	90/104 (87%)	77/99 (78%)	0.10
Annular Abscess	81/203 (40%)	37/104 (36%)	44/99 (44%)	0.2
Fistula	9/203 (4.4%)	4/104 (3.8%)	5/99 (5.1%)	0.7
LVEF	57.9 (8.2)	57.1 (8.0)	58.6 (8.5)	0.2
PAPs > 35 mmHg	42/203 (21%)	23/104 (22%)	19/99 (19%)	0.6
Neurological Event	53/203 (26%)	26/104 (25%)	27/99 (27%)	0.7
Splenic Abscess	31/203 (15%)	17/104 (16%)	14/99 (14%)	0.7

Values are expressed as mean (standard deviation), median [first interquartile, third interquartile] or number (percentage). * Delay was defined as the time interval between the initiation of targeted antibiotic therapy and the date of surgery. BSA: Body Surface Area; BMI: Body Mass Index; COPD: Chronic Obstructive Pulmonary Disease; eGFR: Estimated Glomerular Filtration Rate; NYHA: New York Heart Association; LVEF: Left Ventricular Ejection Fraction; PAPS: Pulmonary Artery Pressure Systolic. Units: Age in years; BSA in m^2^; BMI in kg/m^2^; eGFR in mL/min/1.73 m^2^; LVEF in %. “Aortic Valve (native)” denotes native-valve endocarditis; the remaining patients had prosthetic aortic valve endocarditis.

**Table 2:** Operative data.

Characteristic	Overall, N = 203	Early, N = 104	Late, N = 99	*p*-Value
EuroSCORE II, %	7.3 [3.9, 13.5]	7.1 [3.4, 12.8]	7.4 [4.0, 16.5]	0.4
iAVR	170/203 (84%)	91/104 (88%)	79/99 (80%)	0.14
Homograft	12/203 (5.9%)	3/104 (2.9%)	9/99 (9.1%)	0.061
Bentall	17/203 (8.4%)	8/104 (7.7%)	9/99 (9.1%)	0.7
AV Plasty	4/203 (2.0%)	2/104 (1.9%)	2/99 (2.0%)	>0.9
CABG	11/203 (5.4%)	6/104 (5.8%)	5/99 (5.1%)	0.8
Mitral surgery	50/203 (25%)	28/104 (27%)	22/99 (22%)	0.4
Other surgeries	24/203 (12%)	10/104 (9.6%)	14/99 (14%)	0.3
CPB Time (minutes)	135.0 [99.5, 196.0]	126.5 [97.0, 196.5]	141.0 [102.5, 195.5]	0.3
ACC Time (minutes)	108.0 [80.5, 151.5]	104.0 [76.8, 149.8]	116.0 [83.0, 153.0]	0.3

Values are expressed as median [first interquartile, third interquartile] or number (percentage). EuroSCORE: European System for Cardiac Operative Risk Evaluation; iAVR: Isolated Aortic Valve Replacement; AV: Aortic Valve; CABG: coronary artery bypass grafting; CPB: Cardiopulmonary Bypass; ACC: Aortic Cross-Clamp.

**Table 3:** Early outcomes.

Characteristic	Overall, N = 203	Early, N = 104	Late, N = 99	*p*-Value
In-hospital outcomes				
Death	32/203 (16%)	16/104 (15%)	16/99 (16%)	0.9
IABP	10/203 (4.9%)	4/104 (3.8%)	6/99 (6.1%)	0.5
ECMO	13/203 (6.4%)	8/104 (7.7%)	5/99 (5.1%)	0.4
MAV > 48 h	32/203 (16%)	14/104 (13%)	18/99 (18%)	0.4
Bleeding requiring surgical revision	5/203 (2.5%)	4/104 (3.8%)	1/99 (1.0%)	0.4
Post-operative AF	58/203 (29%)	24/104 (23%)	34/99 (34%)	0.076
Post-operative Stroke	3/203 (1.5%)	1/104 (1.0%)	2/99 (2.0%)	0.6
KDIGO				0.4
No Renal impairment	138/203 (68%)	71/104 (68%)	67/99 (68%)	
Stage 1	22/203 (11%)	13/104 (13%)	9/99 (9.1%)	
Stage 2	22/203 (11%)	8/104 (7.7%)	14/99 (14%)	
Stage 3	21/203 (10%)	12/104 (12%)	9/99 (9.1%)	
Blood Transfusion	130/203 (64%)	59/104 (57%)	71/99 (72%)	0.026
ICU Stay (days)	3.5 (5.5)	2.7 (5.1)	4.3 (5.9)	0.003
Hospital length of stay (days)	21.4 (16.3)	21.3 (17.6)	21.4 (14.9)	0.6
30-day outcomes				
Definite PMK	27/203 (13%)	9/104 (8.7%)	18/99 (18%)	0.046
Re-hospitalization	28/203 (14%)	15/104 (14%)	13/99 (13%)	0.9
Sternal Wound Infection	8/203 (3.9%)	5/104 (4.8%)	3/99 (3.0%)	0.7

Values are expressed as mean (standard deviation) or number (percentage). IABP: Intra-Aortic Balloon Pump; ECMO: Extracorporeal Membrane Oxygenation; MAV: Mechanical Assisted Ventilation; AF: Atrial Fibrillation; KDIGO: Kidney Disease: Improving Global Outcomes (classification for acute kidney injury); ICU: Intensive Care Unit; PMK: Permanent Pacemaker.

**Table 4:** Logistic regression.

	OR	Lower	Upper	*p*-Value	VIF
Group strategy (Late)	2.511	1.073	6.207	**0.034**	1.045
Center					1.076
CHU Mondor	Ref.	Ref.	Ref.	Ref.	
H. CIVILI	0.471	0.116	1.511	0.217	
POLIAMBULANZA	1.068	0.186	4.205	0.932	
eGFR	1.008	0.995	1.021	0.214	1.094
Aortic valve/Prosthesis	1.292	0.492	3.266	0.595	1.161
Age	1.003	0.968	1.042	0.880	1.082
Male Sex	2.062	0.682	8.319	0.214	1.025
EuroSCORE II	1.031	0.992	1.072	0.118	1.292

OR: odds ratio; VIF: variance inflation factor.

**Table 5:** Late outcomes.

Characteristic	Overall, N = 171	Early, N = 88	Late, N = 83	OR, 95% CI	*p*-Value
Total REDO	16/171 (9.4%)	9/88 (10%)	7/83 (8.4%)	0.81, 0.29–2.28	0.7
IE-related REDO	9/171 (5.3%)	5/88 (5.7%)	4/83 (4.8%)	0.84, 0.22–3.24	>0.9
Not operated IE	12/171 (7.0%)	6/88 (6.8%)	6/83 (7.2%)	1.06, 0.33–3.44	>0.9
Total IE relapse	21/171 (12%)	11/88 (13%)	10/83 (12%)	0.96, 0.38–2.39	>0.9
Stroke	5/171 (2.9%)	2/88 (2.3%)	3/83 (3.6%)	1.61, 0.26–9.90	0.7

IE: infective endocarditis. Odds ratios (OR) were calculated with the Woolf logit method. Values are expressed as numbers (percentages).

## Data Availability

The raw data supporting the conclusions of this article will be made available by the authors on request.

## Abbreviations

The following abbreviations are used in this manuscript:

IE	Infective Endocarditis
PMK/PPM	Permanent Pacemaker
ECMO	Extracorporeal Membrane Oxygenation
IABP	Intra-Aortic Balloon Pump
ICU	Intensive Care Unit
KDIGO	Kidney Disease: Improving Global Outcomes
AF	Atrial Fibrillation
MAV	Mechanical Assisted Ventilation
eGFR	Estimated Glomerular Filtration Rate
NYHA	New York Heart Association
LVEF	Left Ventricular Ejection Fraction
PAPs	Pulmonary Artery Systolic Pressure
COPD	Chronic Obstructive Pulmonary Disease
BMI	Body Mass Index
BSA	Body Surface Area
CABG	Coronary Artery Bypass Grafting
CPB	Cardiopulmonary Bypass
ACC	Aortic Cross-Clamp
CT	Computed Tomography
PET	Positron Emission Tomography
MRI	Magnetic Resonance Imaging
AVR	Aortic Valve Replacement
